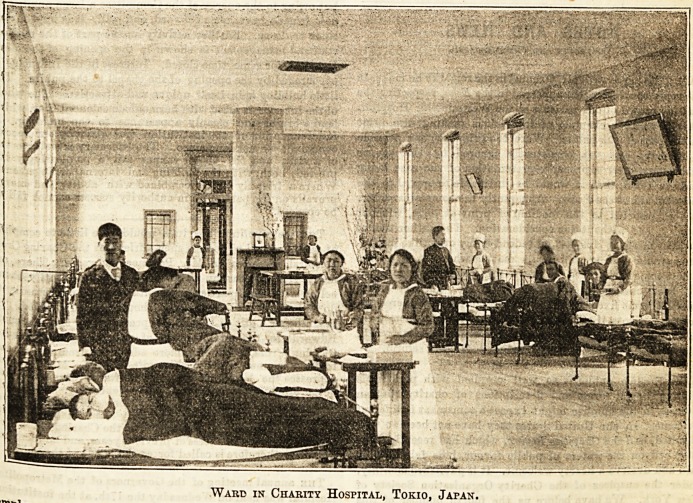# Letters from Far Japan

**Published:** 1892-02-27

**Authors:** Ernest Hart


### LETTERS FROM FAR JAPAN.

By Mrs. ERNEST HART.

### II.—HOSPITALS AND MEDICAL EDUCATION.— PART II.

THE College of Science of the University of Tokio astonishes all European visitors, except those who are acquainted with the enormous strides made by young Japan in scientific studies during the past few years. The character of the Japanese eminently inclines them to patient research, to making elaborate and delicate drawings of microscopical objects, and to close observation of the minute, and it is therefore not surprising to learn that in biology and embryology the researches of the Japanese have already excited much attention, although, as their papers are generally published in German, their work is better known and appreciated in Germany than in England. A marine biological station has been established at Misaki, in the Bay of Tokio, where researches on marine animals are carried on in the laboratory. The professors in the College of Science are, with the exception of Dr. Divers, Professor of Chemistry, all Japanese, who have taken their degrees in London, Cambridge, Paris, or the German universities.

I spent some time in the zoological laboratory of the university, and inspected each student's work. Most of them spoke English, and explained to me with the greatest courtesy and kindness the object of their studies, and showed me their exquisite drawings and beautiful microscopic preparations. The subject of many of the researches then being undertaken was embryological. Some water-colour paintings Mr. Mitsukuri, the Professor of Zoology, showed me of insects and butterflies were more wonderful and realistic than anything I have ever seen in Europe, the very powder on the butterflies' wings being given by means of the most delicate mastery of the technique of painting. Students in the science college, as in that of medicine, are so eager to learn, so anxious not to be behind Europeans in their acquisition of both old and new knowledge, that their eagerness has to be controlled and the hours of study limited. The number of students in this college is small, under one hundred, but it is stated that the demand for graduates in science is larger than the university supplies.

There is a patriotic desire on the part of the professors to disseminate scientific knowledge among the people, and no less than four popular magazines of science are published in the Japanese language. One treats of zoological subjects, another of botanical, a third is a geographical journal, and the fourth is more general, and corresponds to our "Nature." These papers are published monthly, and cost 4d. (10 sen.) a number, which fee only covers the expenses of printing, the literary matter being furnished gratis. The journals are fully illustrated in the European style. It is strange to see in them Latin scientific names and Roman letters sandwiched in between the Japanese type.

The University of Tokio is the crown of the educational system of Japan. The medical and scientific education to be obtained there is excellent, and the degrees granted are worthy of ranking with those of European universities. Not all Japanese students can afford, however, to come up to Tokio to study, and in order to meet the demand for doctors at a time when the old state of things had crumbled away, and the new was not yet organised, a number of private medical schools sprang into existence. These schools, of which there are 29, were about 20 years ago brought under the control of the Department of Education. They are divided into first and second class. In the former a four year's course of study is given, in the latter only three. In many of these schools the opportunities for study are inadequate, and as they are competing bodies for the granting of diplomas to practice, their tendency is to cheapen and degrade medical education. The Japanese are, however, alive to this danger, and they have been urged by Mr. Hart to guard against it by appointing such a body as the General Medical Council armed with similar powers.

There are few hospitals in Japan organised like our English hospitals, that is, free institutions supported by charity, where the poor may be sheltered and cared for, and where trained women devote themselves to the service of suffering. There is, however, one such hospital at Tokio, which it is a real pleasure to visit, namely, the Charity Hospital at Shiba. It is at Shiba that old and new Japan meet, if indeed they do not clash. On one side of the road are the stately and gorgeous Shiba Temples, sacred to the memory and manes of the powerful Tokugawa Shoguns. Standing amid spacious gardens of giant forest trees, these splendid relics of a near yet seemingly remote feudalism excite the keenest admiration by their elaborate architecture, their gorgeous gold and red lac, their intricate carvings of dragons and birds, their richly-chased brass clamps and clasps, their antique paintings and fine damasks. They testify to the Japanese love of hero-worship, and to their belief in greatness as a divinity. But the founded. It is extensive, but simple and complete in all its appointments. Dr. Takagi told me that the Empress was deeply interested in the Charity Hospital, and supported it liberally out of her private purse. Every month she pays it a visit, and goes round to each patient, enquires the nature of his complaint, and says a few words to him or her of sympathy and encouragement. The building is larger than is at present required, and there is room for many more wards when the funds are forthcoming from the charitable for their maintenance.

When I was at the Charity Hospital, Koch's tuberculine had just been received from Europe, and experiments were being made with it with all the early enthusiastic belief in a new remedy. I saw two cases of tubercular disease of the hip joint and the knee, with extensive sinuses, both of which had received remarkable relief from the injection of tuberculine. The sinuses had healed and closed, and the children, both of whom had been in bed for several weeks, could rise, get up, and walk with comparative ease.

Surgical operations are very successful in Japan, and the healing process is rapid, owing probably to the abstinence of

WARD IN CHARITY HOSPITAL, TOKIO, JAPAN.

temples are silent, the gardens are deserted, the stone lamps remain unlit and untrimmed, and no longer stately processions of daimios and priests pass up the flagged pathways, once sacred to their footsteps alone, but now trod by the vulgar public. Japan has turned her mind to other things. She has ceased to worship the dead past, and strains forward to the living future. Leave the splendid and deserted temples, cross the road, and enter the hospital. You might fancy yourself in London. The long wards are spotlessly clean; each patient lies on a low iron bedstead, and in clean sheets, English fashion. The Japanese house surgeon is busy attending to his cases, and the Japanese nurses with their black hair, gathered under a mob-cap, and their kimonos hidden by a broad white apron, are engaged about the wards. The head nurse comes forward to welcome us; she speaks English well, and tells me she was trained at University College Hospital, in London. The picture represents one of the pleasant cheerful wards of the Charity Hospital. Dr. Takagi, the chief physician, who was educated at St. Thomas's, and speaks English like an Englishman, was my guide. He is naturally proud of the hospital, as it has been through his exertions that it has been the people from alcohol and their not being flesh eaters. The Japanese are unsusceptible to scarlet fever, and small-pox, which was at one time rife among them, has been checked and almost exterminated by voluntary vaccination. The mortality of Tokio, a vast city which covers 100 square miles of ground, is only 20 per 1,000. The infant mortality is high, owing to the too early exposure of new-born children. It is not unusual to see a week-old baby strapped on the back of a child of about eight, and sent out to be jumbled about as its infant nurse plays and romps with other children in the street. The Japanese are, like the Chinese, subject to a peculiar disease called kakké, in which the patient is the victim of an increasing lethargy and weakness, and a slow degeneration of the nervous system. It is said to be due to eating rice, which is the staple food of the country, and can be ameliorated, if not cured, by a change of diet.

Nothing could exceed the kindness, the courtesy, and the genial hospitality with which my husband and I were received in Japan by the medical profession, but I must defer, I think, to another letter, some account of Japanese medical banquets, which are unique.

## Figures and Tables

**Figure f1:**